# Deep learning-driven MRI trigeminal nerve segmentation with SEVB-net

**DOI:** 10.3389/fnins.2023.1265032

**Published:** 2023-10-18

**Authors:** Chuan Zhang, Man Li, Zheng Luo, Ruhui Xiao, Bing Li, Jing Shi, Chen Zeng, BaiJinTao Sun, Xiaoxue Xu, Hanfeng Yang

**Affiliations:** ^1^The First Affiliated Hospital, Jinan University, Guangzhou, China; ^2^Department of Radiology, Affiliated Hospital of North Sichuan Medical College, Nanchong, China; ^3^Shanghai United Imaging Intelligence, Co., Ltd., Shanghai, China

**Keywords:** trigeminal neuralgia, trigeminal nerve, deep learning, automatic segmentation, magnetic resonance imaging

## Abstract

**Purpose:**

Trigeminal neuralgia (TN) poses significant challenges in its diagnosis and treatment due to its extreme pain. Magnetic resonance imaging (MRI) plays a crucial role in diagnosing TN and understanding its pathogenesis. Manual delineation of the trigeminal nerve in volumetric images is time-consuming and subjective. This study introduces a Squeeze and Excitation with BottleNeck V-Net (SEVB-Net), a novel approach for the automatic segmentation of the trigeminal nerve in three-dimensional T2 MRI volumes.

**Methods:**

We enrolled 88 patients with trigeminal neuralgia and 99 healthy volunteers, dividing them into training and testing groups. The SEVB-Net was designed for end-to-end training, taking three-dimensional T2 images as input and producing a segmentation volume of the same size. We assessed the performance of the basic V-Net, nnUNet, and SEVB-Net models by calculating the Dice similarity coefficient (DSC), sensitivity, precision, and network complexity. Additionally, we used the Mann–Whitney U test to compare the time required for manual segmentation and automatic segmentation with manual modification.

**Results:**

In the testing group, the experimental results demonstrated that the proposed method achieved state-of-the-art performance. SEVB-Net combined with the *ωDoubleLoss* loss function achieved a DSC ranging from 0.6070 to 0.7923. SEVB-Net combined with the *ωDoubleLoss* method and nnUNet combined with the *DoubleLoss* method, achieved DSC, sensitivity, and precision values exceeding 0.7. However, SEVB-Net significantly reduced the number of parameters (2.20 M), memory consumption (11.41 MB), and model size (17.02 MB), resulting in improved computation and forward time compared with nnUNet. The difference in average time between manual segmentation and automatic segmentation with manual modification for both radiologists was statistically significant (*p* < 0.001).

**Conclusion:**

The experimental results demonstrate that the proposed method can automatically segment the root and three main branches of the trigeminal nerve in three-dimensional T2 images. SEVB-Net, compared with the basic V-Net model, showed improved segmentation performance and achieved a level similar to nnUNet. The segmentation volumes of both SEVB-Net and nnUNet aligned with expert annotations but SEVB-Net displayed a more lightweight feature.

## 1. Introduction

Trigeminal neuralgia is a debilitating neuropathic pain condition that affects psychological, physical, and social needs, such as touching the face, talking, eating, and drinking (Bendtsen et al., 2020). This mental disorder is correlated with a poor quality of life and, in some cases, even a risk of suicide (Obermann, 2019). Clinical diagnosis of trigeminal neuralgia relies on three main criteria: pain localized to the territory of one or more divisions of the trigeminal nerve; paroxysms of intense and brief pain (<1 s to 2 min, but usually a few seconds) described as “shock” or “electric sensation”; and pain triggered by innocuous stimuli on the face or intraoral trigeminal territory (Bendtsen et al., 2019).

**Figure 1:**
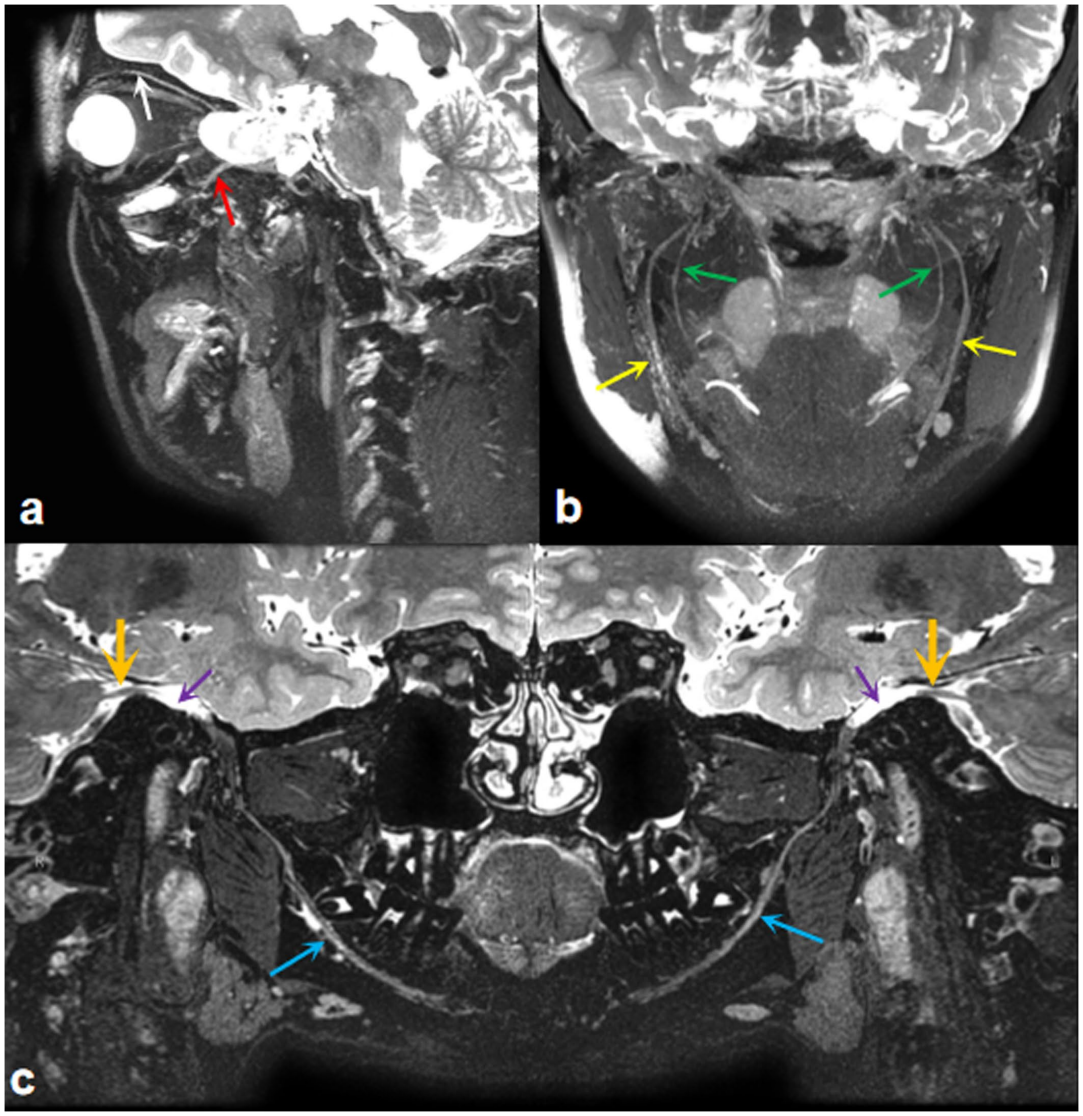
MRI imaging of the trigeminal nerve. **(A)** A three-dimensional T2WI-CUBE fs reconstruction image displays the ocular nerve (white arrow) and maxillary nerve (red arrow). **(B)** A three-dimensional T2WI-CUBE fs reconstruction image shows the branches of the mandibular nerve, inferior alveolar nerve (green arrows), and lingual nerve (yellow arrows). **(C)** A three-dimensional T2WI-CUBE fs reconstruction image provides a comprehensive view of the mandibular branch of the trigeminal nerve, including the trigeminal nerve itself (orange arrows), Meckel cavity (purple arrows), and mandibular nerve (blue arrows).

MR neurography (MRN), dedicated to imaging peripheral nerves, provides a detailed map of neuromuscular anatomy and offers a non-invasive view of intraneural architecture in multiple orthogonal planes (Kim et al., 2023). Furthermore, high-resolution MRI now provides exquisite anatomic detail, enabling radiologists to examine nearly the entire course of the trigeminal nerve, from its nuclei in the brainstem to the distal branches of its three main divisions: the ophthalmic, maxillary, and mandibular nerves (Seeburg et al., 2016). Our team has acquired expertise in utilizing MRI for comprehensive trigeminal nerve imaging, as illustrated in Figure 1. Given the complex course of the trigeminal nerve, reconstructing its peripheral branches is challenging and time-consuming. Therefore, achieving accurate manual delineation and reconstruction requires radiologists with advanced anatomical knowledge and proficient skills. Additionally, it is desirable to develop a trigeminal nerve segmentation model using deep learning methods to enhance clinical efficiency.

Since the introduction of artificial intelligence (AI), deep learning technology has consistently advanced (Aggarwal et al., 2021) demonstrating excellent performance in image analysis using convolutional neural networks (CNNs) (Alzubaidi et al., 2021a). This encompasses object detection, object classification, and object segmentation (Yang and Yu, 2021), all of which contribute to medical diagnosis by enhancing imaging analysis and evaluation (Hwang et al., 2019).

Previously, the statistical shape model (SSM) described by Abdolali et al. was used for automatic segmentation of the inferior alveolar nerve (Abdolali et al., 2016). Lim et al. reported the use of a customized three-dimensional nnU-Net for image segmentation, serving as a fast, accurate, and robust clinical tool for delineating the location of the inferior alveolar nerve (Lim et al., 2021). Lin et al. employed CS2Net to approximately segment the nerves and blood vessels in the trigeminal cistern segment and then refined the boundaries of nerves and blood vessels using three-dimensional UNet, resulting in successful segmentation (Lin et al., 2021). However, there are no existing reports on an artificial intelligence segmentation model for the three branches of the trigeminal nerve.

In this study, our aim was to develop a V-Net-based fully automated framework for the segmentation of the trigeminal nerve using MR imaging. The results show that, in comparison with the basic V-Net, our optimized deep learning algorithm, SEVB-Net, demonstrates good convergence, improved performance in terms of DSC and sensitivity, smoother results than manual segmentation, and superior segmentation performance.

## 2. Methods

### 2.1. Patient data collection

#### 2.1.1. Inclusion and exclusion criteria

We analyzed data obtained from a consecutive series of 232 subjects who underwent MRN at the hospital between January 2020 and December 2022. We excluded 42 subjects due to poor image quality, making it difficult to identify the trigeminal nerve. These data did not clearly depict the nerve, and manual segmentation resulted in poor accuracy, rendering them unsuitable for clinical practice. Additionally, we excluded three subjects in whom manual segmentation did not correspond to the coordinates of the original image. As a result, images from 187 subjects were used in this study, and they were divided into the training (*n* = 152) and testing (*n* = 35) groups.

#### 2.1.2. Image acquisition

To obtain MR neurograms, we utilized magnetic resonance imaging (MRI) scanners, specifically the uMR 790 3.0 T, GE Discovery 750 3.0 T, Magnetom Aera 1.5 T, and Magnetom Skyra 3.0 T, for data acquisition. The scanning sequences for capturing images of the trigeminal nerve are detailed in Table 1. The images of 187 subjects used in this study were sourced from these four scanners: 58 from the uMR 790 3.0 T, 71 from the GE Discovery 750 3.0 T, 32 from the Magnetom Aera 1.5 T, and 26 from the Magnetom Skyra 3.0 T.

**Table 1:** MR scanner and MR neurography protocol.

MR scanner	Tesla (T)	Sequence	TR/TE (ms)	Thickness/Spacing (mm)	Slices	FOV (cm^2^)	Matrix	Flip angle	NEX	Frequency coded	Bandwidth (Hz)	Acquisition time (min)
GE Discovery750	3.0	OCor T2WI CUBE fs	2,500/Maximum	1.0/0	180	18.0 × 18.0	256 × 256	90°	1	S/I	625	7.46
uMR 790	3.0	STIR-MX3D-Cor	4000/174	0.5	224	18 × 24.8	240 × 174	79°	1	S/I	750	7.20
Magnetom Aera	1.5	STIR-SPACE-Cor	3000/225	1.0	128	25.6 × 25.6	256 × 218	120	1	S/I	558	7.59
Magnetom Skyra	3.0	STIR-SPACE-Cor	2500/296	1.0	144	17.5 × 20	256 × 224	120	1	S/I	514	8.10

To ensure an even distribution of scanning protocols in both the training and testing datasets, we randomly divided the data from each scanner according to an 80:20 ratio. In the final dataset, the data quantities for the four scanning protocols were as follows: 47, 58, 26, and 21 in the training set (*n* = 152) and 11,13,6, and 5 in the testing set (*n* = 35).

### 2.2. Manual segmentation

Manual segmentation served as the ground truth in this study, and all manual segmentations were conducted by two radiologists, C and D, each possessing more than 10 years of experience in neuroimaging diagnosis. The 3D Slicer software was employed for pre-processing and manual segmentation, extracting a schematic diagram of the trigeminal nerve from the brainstem to the periphery for each subject. The procedure was as follows:

First, we imported and loaded the 3D-MX STIR images from the Picture Archiving and Communication System (PACS) into 3D Slicer. Then, the images were reformatted and adjusted to display the nerve as clearly as possible. The editor tool in 3D Slicer was used to segment the intracranial and extracranial segments of the trigeminal nerve and its branches (ophthalmic, maxillary, and mandibular nerves) in axial and coronal positions, respectively.

The segmentation accuracy for the trigeminal nerve was subjectively evaluated by two medical experts, Prof. A and Prof. B, each with more than 20 years of experience in neuroimaging diagnosis. The evaluation criteria adopted three levels of scoring: 0, poor (unusable in clinical practice, not included in the study, as mentioned earlier); 1, acceptable (minor revisions needed, subjects included in the study after revision); and 2, good (nearly no revisions needed, subjects directly included in the study).

### 2.3. Data pre-processing

In this study, two types of data pre-processing were employed. The first type was conducted before training and included bias field correction and region of interest (ROI) division based on connected domains.

#### 2.3.1. Bias field correction

**Figure 2:**
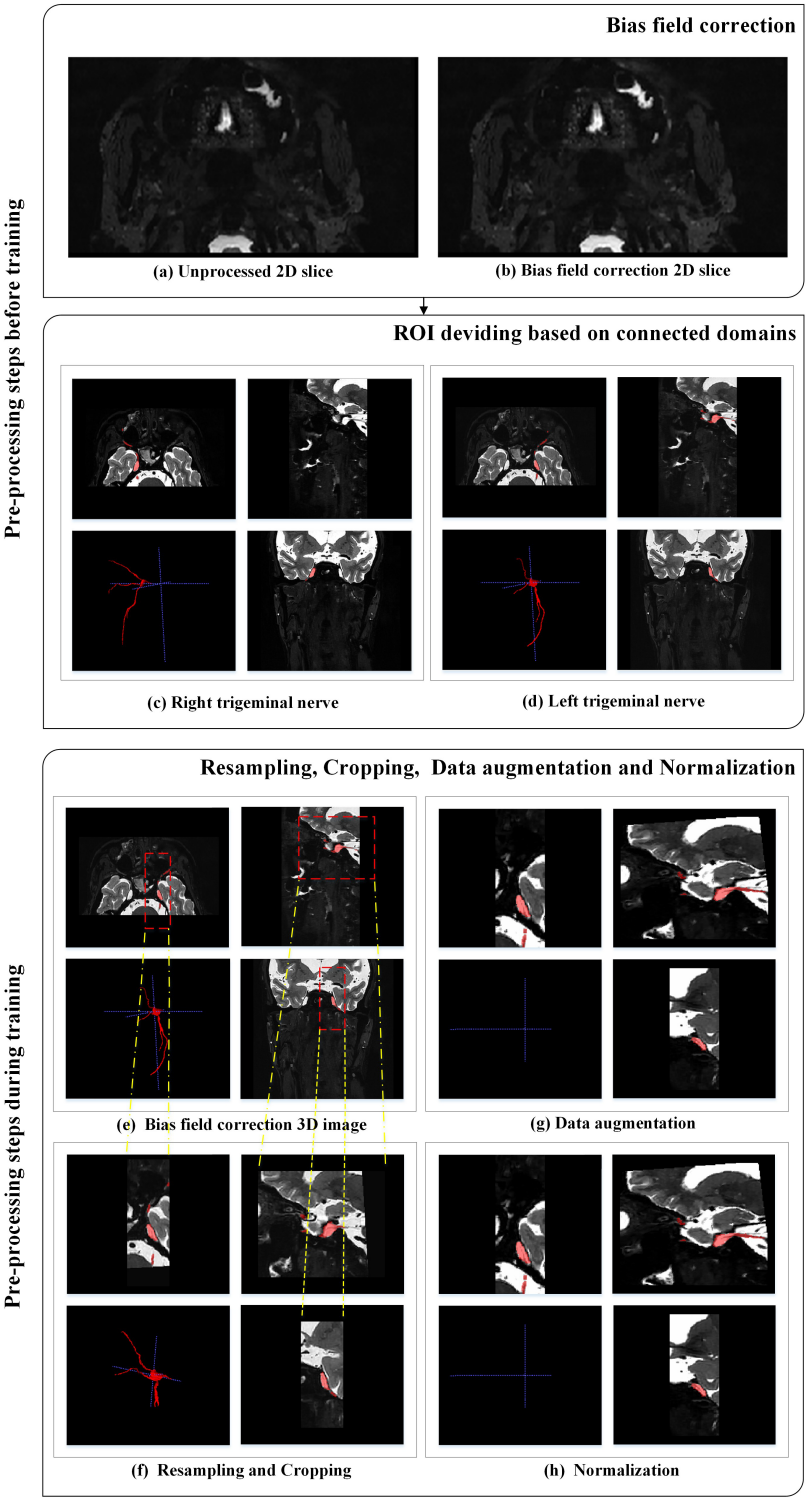
Pre-processing steps applied to each MRI case.

Various factors such as different scanners, scanning schemes, and acquisition artifacts can result in an uneven display intensity of MR images during visualization, causing variations in the intensity values of the same tissue across the entire MR image (Ullah et al., 2021). This variation in intensity is referred to as the bias field (Bernal et al., 2019), which can affect the quality of the acquired MR images. To address this bias field distortion, we applied the N4ITK bias field correction algorithm, a commonly used strategy in the literature, proposed by Tustison et al. (2010), and evaluated the publicly available datasets BraTS-2013, BraTS-2015, and BraTS-2018 (Ullah et al., 2021). The N4ITK bias field correction algorithm was applied to all images to mitigate image artifacts and enhance grayscale distribution. Sample images before and after bias field correction are shown in Figures 2A,B.

#### 2.3.2. Region of interest dividing based on connected domain

After radiologists manually segmented the trigeminal nerve regions, these regions were then divided based on connected domains. The original bilateral trigeminal nerves were divided into left and right trigeminal nerves (as shown in Figures 2C,D). This division not only doubled the amount of training data but also reduced GPU memory consumption during training.

The second type of data pre-processing occurred during training.

#### 2.3.3. Resampling and cropping

Medical images often have inconsistent resolutions. To enable the network to accurately learn spatial semantics, all patients were resampled to the median voxel spacing of the dataset (Isensee et al., 2021). We used nearest-neighbor interpolation for both the original image data and the corresponding segmentation mask. During training, we randomly sampled cropped patches of the same size from the image and used them as network input to reduce GPU memory consumption, as shown in Figures 2E,F.

#### 2.3.4. Data augmentation

Data augmentation was applied to the original images (as shown in Figures 2F,G). Data augmentation is crucial in deep learning as it helps generate additional equivalent data to expand the dataset (Chlap et al., 2021). In this study, we employed various data augmentation techniques, including common geometric transformations (such as rotation, scaling, translation, mirror image, and elastic deformation) and color transformations (such as brightness transformation, gamma correction, cover, filling, Gaussian noise transformation, and Gaussian blurring transformation). Increasing the training data is beneficial for improving the model’s generalization ability, and incorporating appropriate noise data can enhance the model’s robustness.

#### 2.3.5. Intensity normalization

The images were adaptively normalized to the range of [−1, 1], as illustrated in Figure 2H. All intensity values within the ROI of the image were collected and scaled to occupy [0.01, 0.99] of these intensity values, and *z*-score normalization was performed based on the minimum and maximum intensity values. Figure 2 illustrates the pre-processing steps applied to each MRI case.

### 2.4. Overall framework for trigeminal nerve segmentation

**Figure 3:**
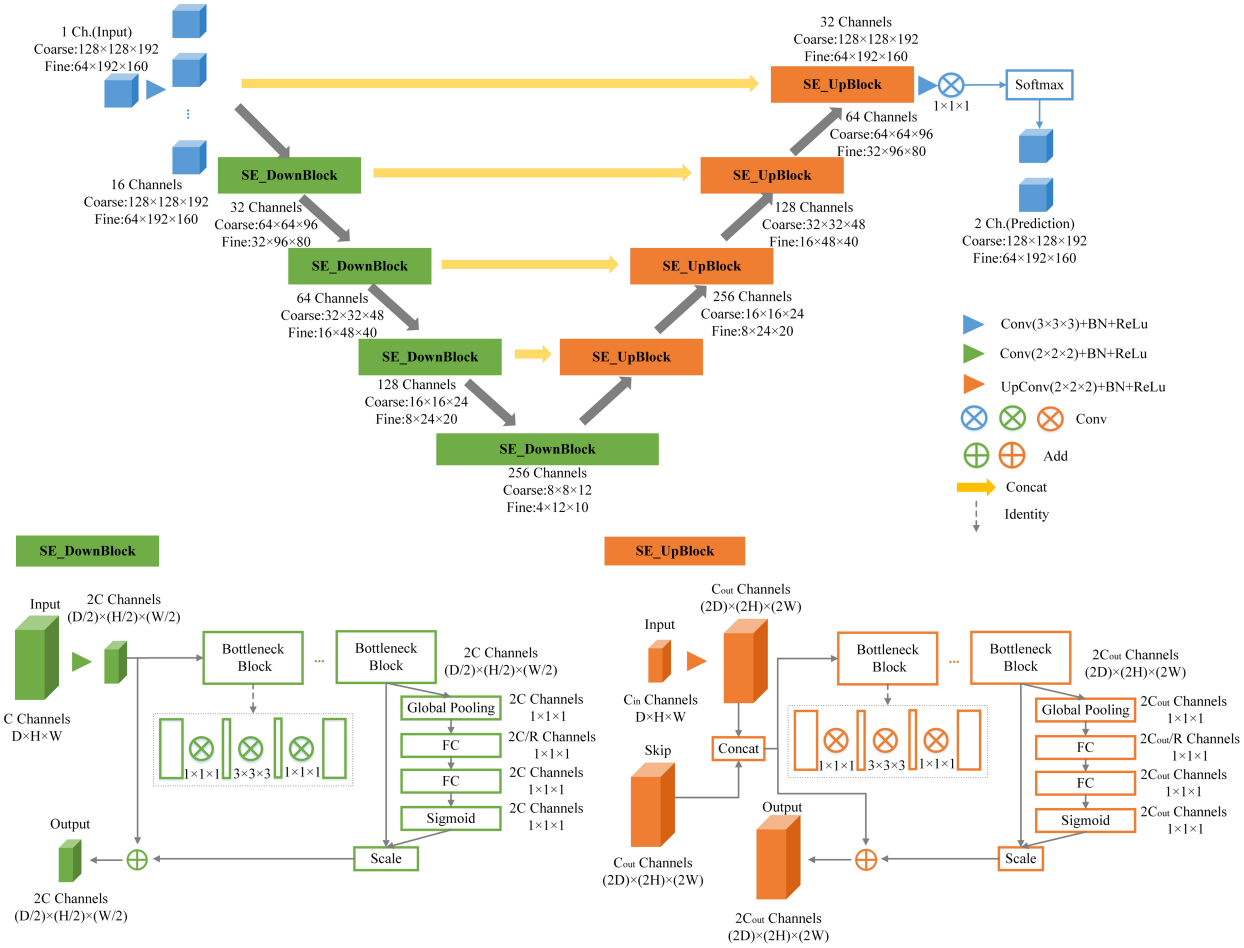
Schematic representation of our network architecture.

This research employed an end-to-end network model for semantic segmentation, independently developed by a dedicated data processing team, utilizing a three-dimensional segmentation scheme. Expanding upon the V-Net network as the baseline, we introduced the BottleNeck and SE-Net components to optimize the V-Net network structure (Milletari et al., 2016). We named the architecture as SEVB-Net, where “SE” stands for SE-Net and “B” stands for bottleneck. As depicted in Figure 3, the bottleneck structure comprises three convolutional layers and replaces the original 3 × 3 × 3 kernel layers (He et al., 2016). Two 1 × 1 × 1 kernel layers are utilized to reduce and increase (recover) the number of channels, respectively, while the central 3 × 3 × 3 kernel layer is employed for feature map processing. This design offers several advantages: (1) It significantly reduces the number of parameters, leading to reduced computational complexity and a smaller model size; (2) after dimension reduction, it enables more effective and intuitive data training and feature extraction. The features following dimension increase are tailored specifically to the current task; and (3) compared with the traditional convolutional structure, it incorporates a shortcut branch that facilitates the transmission of low-level information, thereby enhancing deep and efficient training.

SE-Net adopts a channel-centric perspective and introduces the “Squeeze and Excitation (SE)” module, which enhances accuracy by modeling the correlation between feature channels and strengthening critical features (Hu et al., 2020). The squeeze operation compresses features along the spatial dimension, resulting in a one-dimensional feature matching the number of feature channels. This feature represents the global distribution of responses across the feature channels. The excitation operation models correlations between feature channels through two fully connected layers and assigns weight to each feature channel. These weights signify the importance of each feature channel after feature selection. Finally, the channels are applied to the previous features, completing the recalibration of the original features along the channel dimension. Through this mechanism, the network leverages global information to selectively emphasize informative features while suppressing less useful ones.

### 2.5. Training procedure

**Figure 4:**
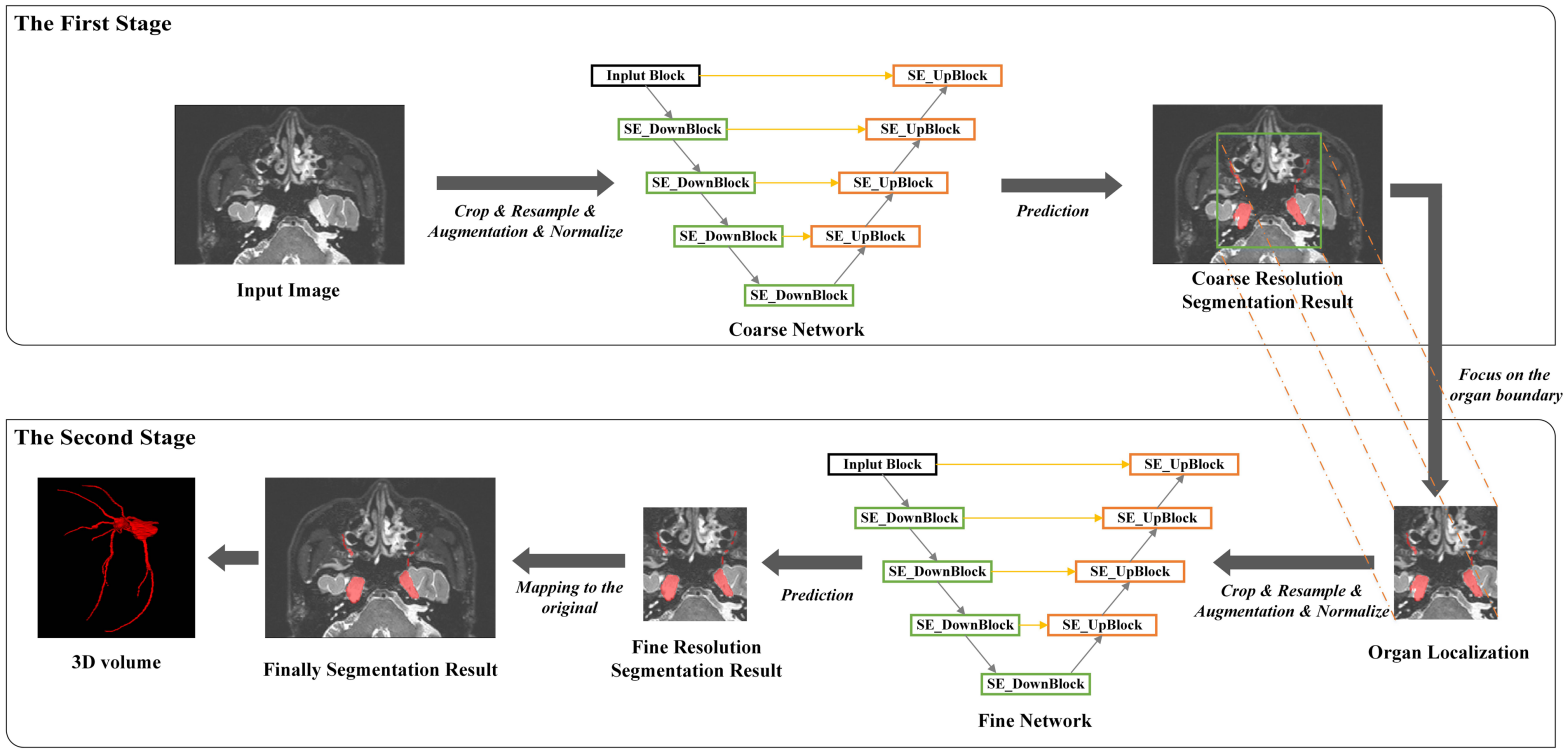
An overview of the multi-resolution network.

There were 152 MR T2 scans with the trigeminal nerve for training and 35 for testing. Images in the training set had a spatial resolution of 0.5 × 0.5 × 0.5 mm. Owing to the large size of three-dimensional medical images, to reduce memory usage, we adopted a multi-resolution strategy to train two SEVB-Nets on different image resolutions (Mu et al., 2019). As illustrated in Figure 4, at the coarse resolution, we trained an SEVB-Net to approximately localize the entire trigeminal nerve region. This was achieved by resampling images to an isotropic spacing of 1 mm and using a fixed input patch size of 128 × 128 × 192, randomly sampled from the entire image domain for training.

Following the localization of the trigeminal nerve region, we trained a fine-resolution SEVB-Net for detailed segmentation. As the trigeminal nerve had already been segmented at the coarse resolution, the fine segmentation model was only trained by resampling images to an isotropic spacing of 0.5 mm and randomly sampling image patches 64 × 192 × 160 in size from the unilateral trigeminal nerve region indicated by the split manual segmentation (as shown in Figures 2C,D) of input images.

During the inference stage, the entire image volume was divided into overlapping subvolumes using a sliding window, and the overlapping parts were combined using Gaussian weighted averaging. This approach allowed SEVB-Net to accurately segment the trigeminal nerve boundaries.

### 2.6. Loss function

Image segmentation based on supervised learning requires a loss function to quantify the error between the predicted segmentation and the manual segmentation during the learning process. This helps in continuously optimizing the segmentation performance. In this study, we employed a multi-loss mixture function to measure the predicted segmentation of the network. Specifically, we used the dice coefficient loss (*L*_dice_) and the focal loss (*L*_focal_) as the loss functions *L*_DoubleLoss_ for network training. The *L*_DoubleLoss_ is defined as:


(1) $$L_{\mathrm{DoubleLoss}}=\omega L_{\mathrm{dice}}+1-\omega L_{\mathrm{focal}}$$

where *ω* is a weight balance parameter between *L*_dice_ (Milletari et al., 2016) and *L*_focal_ (Lin et al., 2020), which is empirically set as *ω* = 0.5.

The dice loss reduces the model’s sensitivity to unbalanced classes by considering the entire situation and focusing primarily on the foreground voxels. The focal loss, on the other hand, addresses pixel-level issues from a micro perspective and complements the dice loss. It guides the dice loss and provides the network with a gradient descent direction when the dice coefficient is zero during training. The dice loss function is defined as:


(2) $$L_{\mathrm{dice}}=1-\frac{1}{c}\overset{C}{\underset{c=1}{\sum }}\frac{2\sum_{i}^{N}p_{c}\left(i\right)g_{c}\left(i\right)}{\sum_{i}^{N}p_{c}{\left(i\right)}^{2}+\sum_{i}^{N}g_{c}{\left(i\right)}^{2}}$$

where the inner summation runs over the *N* voxels in the image domain, *C* represents the number of class labels, *p_c_*(*i*) is the probability of class *c* at voxel *i* predicted by the network, and *g_c_*(*i*) is the binary label indicating whether the label of voxel *i* is class *c*.

Additionally, the *L*_focal_ is defined as:


(3) $$L_{\mathrm{focal}}\left(p_{t}\right)=-\alpha_{t}{\left(1-p_{t}\right)}^{\gamma }\log\left(p_{t}\right)$$

Here, *p*_t_ is the predicted probability, *α* is the class weighting parameter, and *γ* is the modulating factor that shifts the model’s focus toward learning hard negatives during training by down-weighting the easy examples. We define *p*_t_ as (Lin et al., 2020):


(4) $$p_{t}=\{\begin{array}{c} p,\mathrm{if}y=1\\ 1-p,\mathrm{otherwise} \end{array}$$

**Figure 5:**
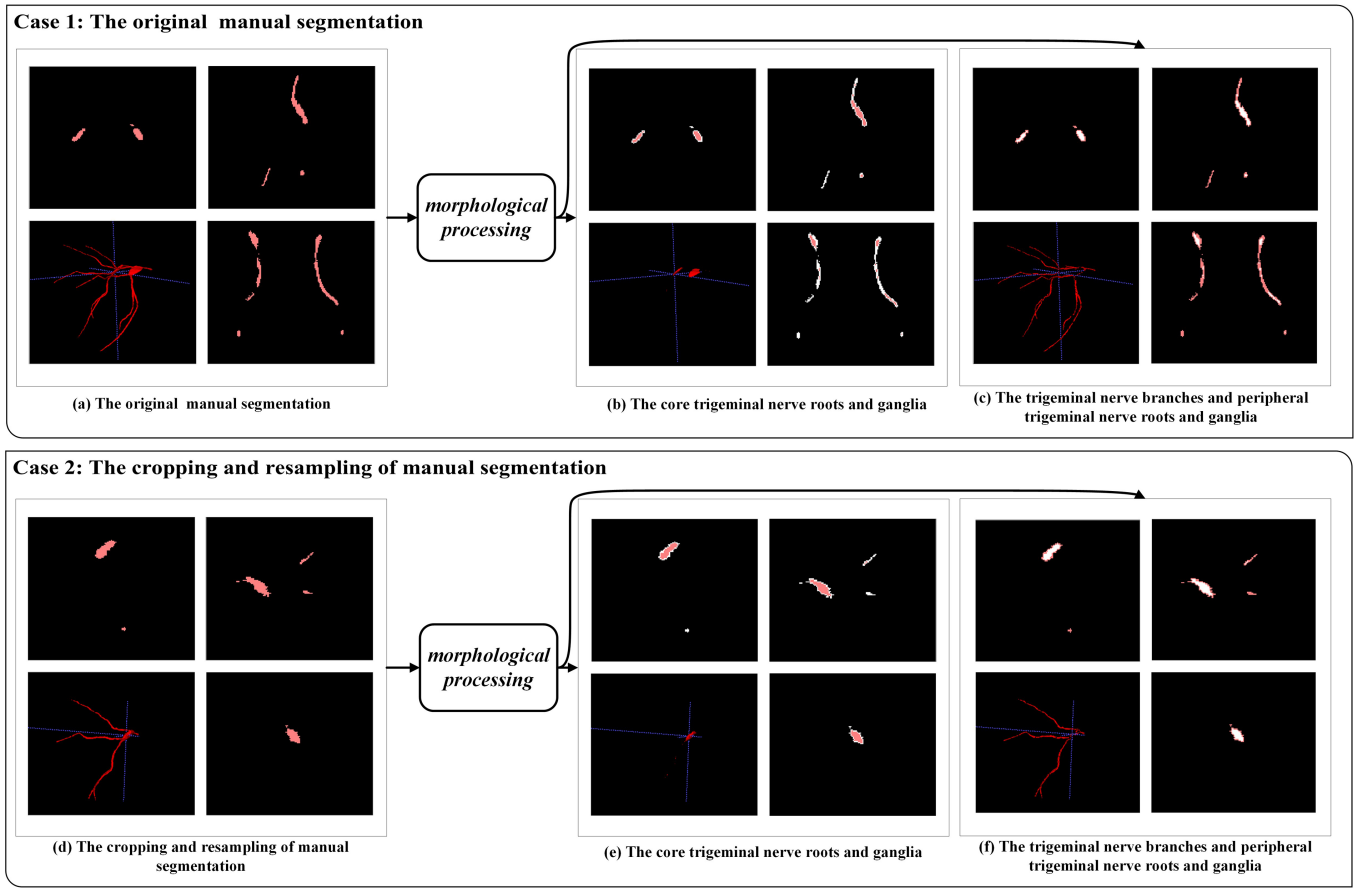
An example of morphological processing. Case 1: morphological processing of the original manual segmentation. Case 2: morphological processing after cropping and resampling of manual segmentation.

In the case of the trigeminal nerve in this study, the segmentation difficulty lay in the fact that, within the same sample, the trigeminal nerve root and ganglion were relatively easy to segment, whereas the trigeminal nerve branches, including the ophthalmic nerve, maxillary nerve, and mandibular nerve, were prone to under-segmentation. To address this limitation, morphological processing was used to approximately separate easily segmented areas from challenging ones. As shown in Figure 5, the trigeminal nerve branches and peripheral trigeminal nerve roots and ganglia were distinguished from the core trigeminal nerve roots and ganglia. Subsequently, we defined the original manual segmentation as Ω, with inside Ω (as shown in Figures 4B,E) and outside Ω (as shown in Figures 4C,F) denoting the above two areas, respectively (Lv et al., 2019). The weighted focal loss was defined as:


(5) $$L_{\omega \mathrm{focal}}\left(\Omega \right)=\lambda_{1}L_{\mathrm{focal}}\left(\mathrm{inside}\;\Omega \right)+\lambda_{2}L_{\mathrm{focal}}\left(\mathrm{outside}\;\Omega \right)$$

where *λ*_1_ and *λ*_2_ are two balancing parameters used to modulate the influence of the inside Ω error and outside Ω error.

Based on these considerations, we used the dice coefficient loss (*L*_dice_) and the weighted focal loss (*L*_ωfocal_) as the final loss function *L*_ωDoubleLoss_ for network training, defined as:


$$L_{\omega \mathrm{DoubleLoss}}=\omega L_{\mathrm{dice}}+\left(1-\omega \right)L_{\mathrm{wfocal}}$$

(6) $$=\omega L_{\mathrm{dice}}+\left(1-\omega \right)\left(\lambda_{1}L_{\mathrm{focal}}\left(\mathrm{inside}\;\Omega \right)+\lambda_{2}L_{\mathrm{focal}}\left(\mathrm{outside}\;\Omega \right)\right)$$

where *ω* is a weight balance parameter between *L*_dice_ and *L*_ωfocal_ and *λ*_1_ and *λ*_2_ are two balancing parameters used to modulate the influence of inside Ω error and outside Ω error.

### 2.7. Comparison of segmentation time between manual and automatic segmentation with manual modification

To evaluate the segmentation efficiency of the deep learning model, we first calculated the time required for manually segmenting 35 test data points and then determined the time required for automatic segmentation with manual modification of the same 35 test data points. The process of manual modification was carried out by two experienced radiologists (C and D), each specializing in segmentation. The scores for manual segmentation and automatic segmentation with manual modification, evaluated by Prof. A and Prof. B, should be up to 3. We used the Mann–Whitney U test to compare the difference in average time needed between manual segmentation and automatic segmentation with manual modification.

## 3. Material and evaluation metrics

### 3.1. Data set characteristics

The characteristics of the subjects in the training and testing groups are summarized in Table 2. Continuous features such as age were compared using ANOVA. Categorical features such as gender and clinical features were compared using the Chi-square method. There were no significant differences among the three groups concerning age, gender, and clinical features.

**Table 2:** Demographic and clinical characteristics of subjects in the training (*n* = 152) and test groups (*n* = 35).

Characteristics	Training group	Testing group	Statistics
Age (year) (mean ± SD)	56.3 ± 9.5	54.6 ± 6.7	*p* = 0.947
Gender (%)			
Male	60 (39.5%)	13 (37.1%)	*p* = 0.799
Female	92 (60.5%)	22 (62.9%)	
Clinical features (%)			
Pain	72 (47.4%)	16 (45.7%)	*p* = 0.860
Normal	80 (52.6%)	19 (54.3%)	

### 3.2. Evaluation metrics

To quantitatively measure the performance of the proposed method, we used the Dice similarity coefficient (also known as F1 score) (Jaffari et al., 2021) to assess the similarity between the segmentation volume and manual segmentation. The evaluation measure is within the range of 0 to 1, with higher coefficient values indicating better segmentation performance. The definition is as follows:


(7) $$DSC\left(F1\mathrm{Score}\right)=\frac{2TP}{2TP+FP+FN}$$

Additionally, the quality of the segmentation volume is typically evaluated using accuracy, precision, sensitivity (also known as recall), and specificity (Cruz-Aceves et al., 2018; Gao et al., 2022). However, there is a significant class imbalance in the samples, as the trigeminal nerve and background pixels are denoted as positive and negative classes, respectively. Accuracy and specificity primarily assess the performance of background pixel segmentation. Therefore, our main focus is on precision and sensitivity (also known as recall), which are two parameters used to evaluate the performance of foreground pixel segmentation. These evaluation parameters are defined as follows:


(8) $$\mathrm{Precision}=\frac{TP}{TP+FP}\mathrm{and}$$

(9) $$S\mathrm{ensitivity}\left(R\mathrm{ecall}\right)=\frac{TP}{TP+FN}$$

where trigeminal nerve and background pixels are denoted as positive and negative classes. TP, TN, FP, and FN represent the pixel counts of true positives, true negatives, false positives, and false negatives, respectively.

## 4. Experimental results

**Figure 6:**
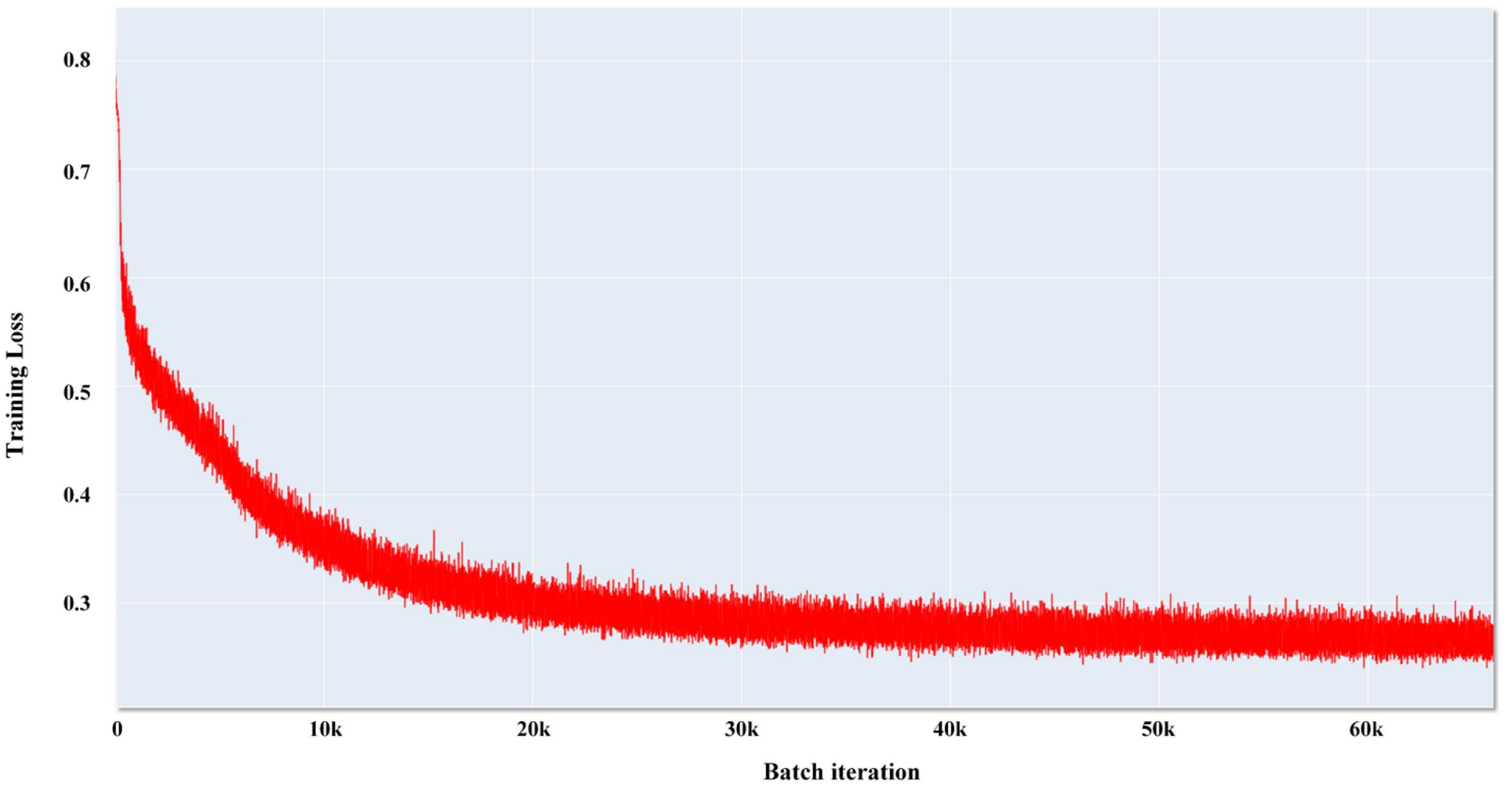
Convergence curve of SEVB-Net combined with the *ωDoubleLoss* method.

We compared the most popular models for medical image segmentation, V-Net, and nnUNet, with SEVB-Net. Simultaneously, we compared the proposed loss function *DoubleLoss* and its variant *ωDoubleLoss* to demonstrate the segmentation performance of the proposed method. The training process adopted a multi-resolution strategy, with the learning rate set to 1e^−4^, the decay rate set to 0.1 every 1,000 epochs, and a maximum of 6,000 epochs. Within these 6,000 epochs, the model with the best performance was selected for evaluation. The training loss of SEVB-Net combined with the *ωDoubleLoss* method is displayed in Figure 6, showing good convergence (Man et al., 2019).

Table 3 summarizes the average Dice similarity coefficient (DSC), sensitivity, and precision of the network direct output results of the four experiments (experiment 1, V-Net combined with the *DoubleLoss* method; experiment 2, SEVB-Net combined with the *DoubleLoss* method; experiment 3, nnUNet combined with the *DoubleLoss* method; and experiment 4, SEVB-Net combined with the *ωDoubleLoss* method) on the testing set (*n* = 35). First, ANOVA was used to determine whether there was a difference between the means of the four experiments. From the results of the first row of each indicator in Table 4, it could be concluded that there was no significant difference in the DSC (*p* = 0.17, *p* > 0.1) between the four experiments, the sensitivity tended to be significantly different (*p* = 0.0794, *p* < 0.1), and there was a significant difference in the precision (*p* = 0.0291, *p* < 0.05). After that, Tukey’s test in multiple comparisons was used to further determine which two means differed from each other, and which did not. Additionally, a post-hoc Benjamini–Hochberg (BH) adjustment was applied to balance the likelihood of false positive and false negative findings. Finally, the effect sizes for individual *t*-tests obtained by Cohen’s *d* were reported. As shown in Table 4, the difference in precision between experiment 2 (SEVB-Net combined with the *DoubleLoss* method) and experiment 3 (nnUNet combined with *DoubleLoss* method) tended to be significantly different (adjusted value of *p* = 0.0972, *p* < 0.1; *d* = 0.7335, *d* > 0.5). A commonly used interpretation is to refer to effect sizes as small (*d* = 0.2), medium (*d* = 0.5), and large (*d* = 0.8) based on benchmarks suggested by Cohen. The individual pairwise comparisons were analyzed in the following subsections.

**Table 3:** Quantitative performance of different algorithms.

SN	Network	Loss function	DSC	DSC range	Sensitivity	Precision
1	V-Net	DoubleLoss	0.6934 ± 0.0471	0.5919 ~ 0.7789	0.6903 ± 0.0534	0.7006 ± 0.0665
2	SEVB-Net	DoubleLoss	0.6984 ± 0.0470	0.6054 ~ 0.7850	0.7260 ± 0.0643	0.6790 ± 0.0688
3	nnUNet	DoubleLoss	0.7170 ± 0.0452	0.6307 ~ 0.8084	0.7105 ± 0.0540	0.7277 ± 0.0638
4	SEVB-Net	ωDoubleLoss	0.7061 ± 0.0445	0.6070 ~ 0.7923	0.7089 ± 0.0521	0.7081 ± 0.0674

**Table 4:** Significance test of quantitative performance of different algorithms.

		Estimate	Std	*F*/*t*	Value of *p*	Adjusted value of *p*	Cohen’s *d*
Dice	–	–	–	1.7000	0.1700	–	–
	(SEVB-Net + *DoubleLoss*) − (V-Net + *DoubleLoss*) == 0	0.0051	0.0111	0.4530	0.9690	0.9690	0.1074
	(nnUNet+*DoubleLoss*) − (V-Net + *DoubleLoss*) == 0	0.0236	0.0111	2.1180	0.1530	0.9180	0.5114
	(SEVB-Net + *ωDoubleLoss*) − (V-Net + *DoubleLoss*) == 0	0.0128	0.0111	1.1450	0.6620	0.9690	0.2786
	(nnUNet + *DoubleLoss*) − (SEVB-Net + *DoubleLoss*) == 0	0.0186	0.0111	1.6650	0.3460	0.9690	0.4024
	(SEVB-Net + *ωDoubleLoss*) − (SEVB-Net + *DoubleLoss*) == 0	0.0077	0.0111	0.6920	0.9000	0.9690	0.1685
	(SEVB-Net + *ωDoubleLoss*) − (nnUNet + *DoubleLo*ss) == 0	−0.0108	0.0111	−0.9730	0.7650	0.9690	0.2418
Sensitivity	–	–	–	2.3070	**0.0794**	–	–
	(SEVB-Net + *DoubleLoss*) − (V-Net + *DoubleLoss*) == 0	0.0357	0.0136	2.6230	0.0473*	0.2838	0.6043
	(nnUNet + *DoubleLoss*) − (V-Net + *DoubleLoss*) == 0	0.0203	0.0136	1.4890	0.4468	0.8034	0.3777
	(SEVB-Net + *ωDoubleLoss*) − (V-Net + *DoubleLoss*) == 0	0.0186	0.0136	1.3650	0.5233	0.8034	0.3527
	(nnUNet + *DoubleLoss*) − (SEVB-Net + *DoubleLoss*) == 0	−0.0154	0.0136	−1.1330	0.6695	0.8034	0.2599
	(SEVB-Net + *ωDoubleLoss*) − (SEVB-Net + *DoubleLoss*) == 0	−0.0171	0.0136	−1.2570	0.5915	0.8034	0.2927
	(SEVB-Net + *ωDoubleLoss*) − (nnUNet + *DoubleLoss*) == 0	−0.0017	0.0136	−0.1240	0.9993	0.9993	0.0318
Precision	–	–	–	3.0950	0.0291*	–	–
	(SEVB-Net + *DoubleLoss*) − (V-Net + *DoubleLoss*) == 0	−0.0216	0.0162	−1.3340	0.5433	0.7453	0.3184
	(nnUNet + *DoubleLoss*) − (V-Net + *DoubleLoss*) == 0	0.0271	0.0162	1.6760	0.3402	0.6804	0.4158
	(SEVB-Net + *ωDoubleLoss*) − (V-Net + *DoubleLoss*) == 0	0.0075	0.0162	0.4660	0.9664	0.9664	0.1124
	(nnUNet + *DoubleLoss*) − (SEVB-Net + *DoubleLoss*) == 0	0.0486	0.0162	3.0100	0.0162*	**0.0972**	0.7335
	(SEVB-Net + *ωDoubleLoss*) − (SEVB-Net + *DoubleLoss*) == 0	0.0291	0.0162	1.7990	0.2783	0.6804	0.4271
	(SEVB-Net + *ωDoubleLoss*) − (nnUNet + *DoubleLoss*) == 0	−0.0196	0.0162	−1.2110	0.6211	0.7453	0.2984

Signif. codes: 0 ‘***’ 0.001 ‘**’ 0.01 ‘*’ 0.05‘.’ 0.1 ‘’ 1. All bold values are less than 0.1, indicating statistical significance.

### 4.1. Comparison of the quantitative performances of different network structures

When comparing experiment 1 (V-Net combined with the *DoubleLoss* method) to experiment 2 (SEVB-Net combined with the *DoubleLoss* method), the performance of SEVB-Net improved in both the DSC and sensitivity but there were no significant differences. Compared with experiment 1 (V-Net combined with the *DoubleLoss* method), experiment 3 (nnUNet combined with the *DoubleLoss* method) showed an improved performance in terms of the DSC, sensitivity, and precision but there were also no significant differences. The difference in precision between experiment 2 (SEVB-Net combined with the *DoubleLoss* method) and experiment 3 (nnUNet combined with the *DoubleLoss* method) tended to be significantly different (adjusted value of *p* = 0.0972, *p* < 0.1; *d* = 0.7335, *d* > 0.5), and the output results of SEVB-Net were not as good as those of nnUNet in terms of precision but there were no significant differences in the DSC and sensitivity.

### 4.2. Comparison of quantitative performance of different loss functions

After using *ωDoubleLoss* instead of *DoubleLoss*, the performance of experiment 4 (SEVB-Net combined with the *ωDoubleLoss* method) was improved compared with experiment 1 (V-Net combined with the *DoubleLoss* method) in terms of the DSC, sensitivity, and precision, but there were no significant differences. When comparing experiment 2 (SEVB-Net combined with the *DoubleLoss* method) with experiment 4 (SEVB-Net combined with the *ωDoubleLoss* method), the network outputs of the *ωDoubleLoss* method compared with the *DoubleLoss* method had an advantage in terms of DSC and precision, but there were no significant differences. There was no significant difference between experiment 3 (nnUNet combined with the *DoubleLoss* method) and experiment 4 (SEVB-Net combined with the *ωDoubleLoss* method), indicating that the trend of significant difference in precision between SEVB-Net and nnUNet was brought closer after using *ωDoubleLoss* instead of *DoubleLoss*.

### 4.3. Overall quantitative performance comparison

**Figure 7:**
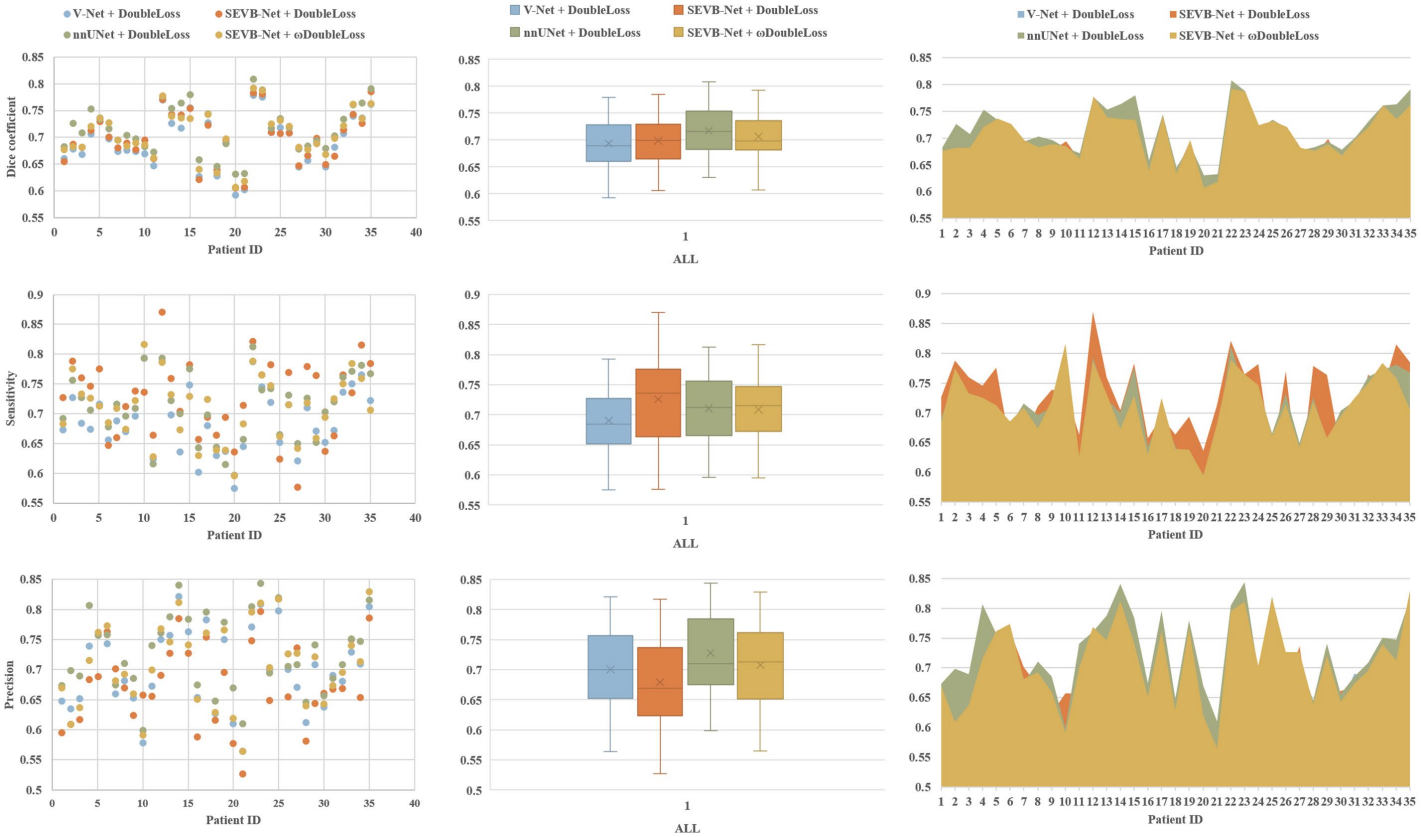
Comparative analysis of the Dice similarity coefficient (DSC), sensitivity, and precision of the three experiments on the testing set.

The rows in Figure 7 represent the three performance metrics of the DSC, sensitivity, and precision, respectively. Each column displays each metric using a scatter plot, box plot, and area plot, further confirming the results of Tables 3, 4. First, regarding the DSC, the highest DSC values for the four experiments were 0.7789, 0.7850, 0.8084, and 0.7923, and the lowest DSC values were 0.5919, 0.6054, 0.6307, and 0.6070, respectively. The highest and lowest DSCs of SEVB-Net combined with the *ωDoubleLoss* method were lower than the nnUNet method but higher than the other two methods. Additionally, SEVB-Net combined with the *DoubleLoss* method had an advantage in sensitivity and nnUNet performed best in precision. Finally, the nnUNet method and SEVB-Net combined with the *ωDoubleLoss* method achieved a DSC, sensitivity, and precision above 0.7, indicating that the segmentation volumes were in agreement with expert annotations.

### 4.4. Comparison of network complexity

We compared the parameters, memory consumption, FLOPS, forward time (averaged over 100 trials on an NVIDIA TITAN RTX), number of convolutional layers, and the coarsest stride of the different network. The number of parameters (2.20 M), memory consumption (11.41 MB), and model size (17.02 MB) of SEVB-Net were significantly reduced, and the computation and forward time were substantially improved compared with nnUNet (the details are listed in Table 5).

**Table 5:** Comparison of parameters, memory consumption, FLOPS, forward time (averaged over 100 trials on an NVIDIA TITAN RTX), number of convolutional layers, and the coarsest stride of different networks.

Network	V-Net	nnUNet	SEVB-Net
Parameters (M)	14.56	45.36	2.20
Memory (MB)	74.20	235.12	11.41
Deployment model size (MB)	55.63	235.12	8.51
Cascade deployment model size (MB)	111.25	–	17.02
FLOPS (GMac)	363.65	1574.83	252.57
Forward time (s)	16.59	60.17	15.42
Convolutional layers	29	32	57
Max stride	16	16	16

FLOPS, floating-point operations per second; GMac, giga multiply-add operations per second.

**Figure 8:**
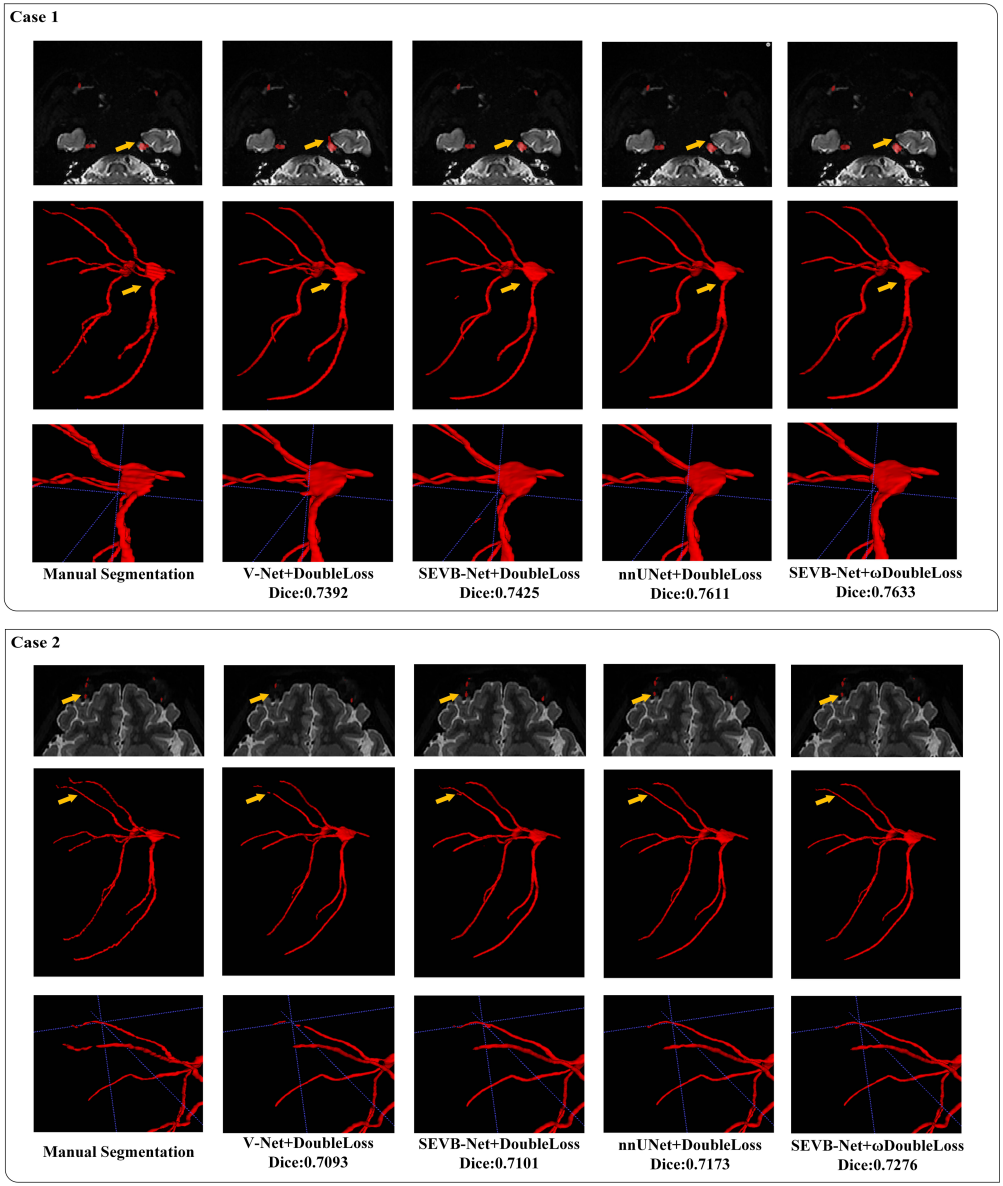
Visualization of the segmentation results.

**Figure 9:**
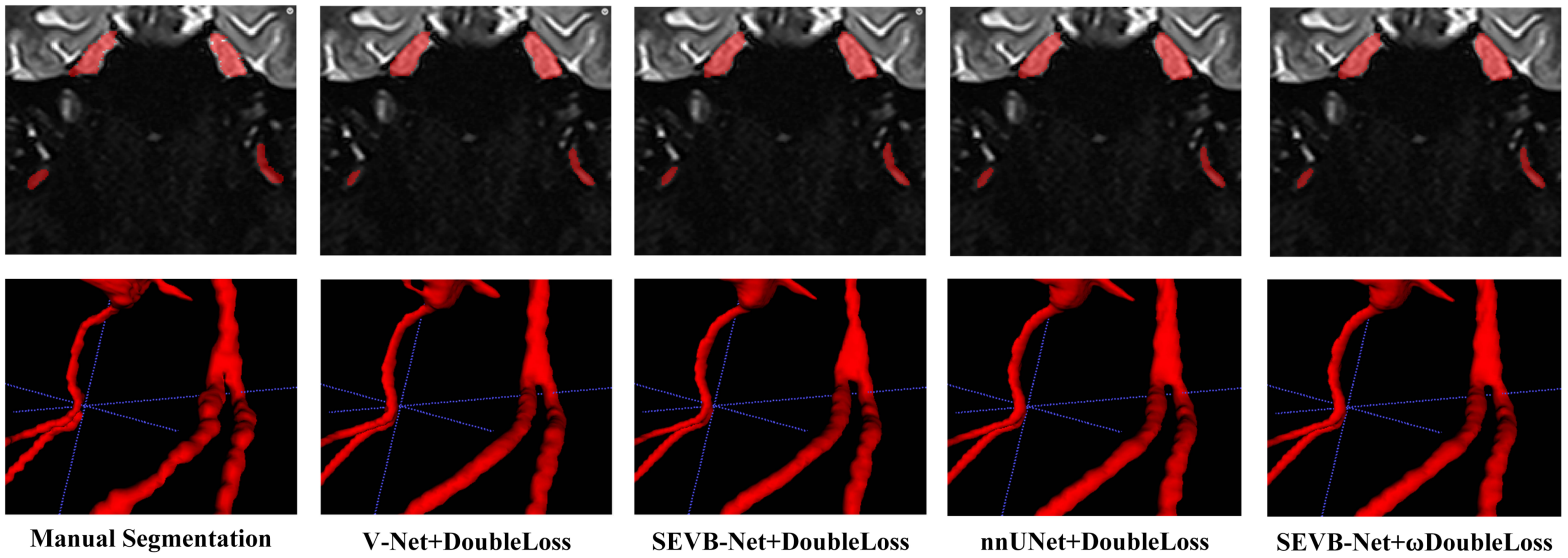
Comparison of the boundaries of segmentation results.

In addition, we performed three-dimensional rendering on the segmentation volumes of the four experiments. Figure 8 displays the rendering results of two test samples. For each case, the first row represents a two-dimensional slice, the second row depicts the three-dimensional rendering of the segmentation volume, and the third row illustrates partial three-dimensional rendering. Upon observation, it was evident that in the current training scale, the segmentation results all exhibited under-segmentation across the four experiments. However, V-Net was more prone to under-segmentation and incorrect segmentation, which SEVB-Net improved to a certain extent. Subsequently, after replacing *DoubleLoss* with *ωDoubleLoss*, the segmentation result became more continuous and accurate, comparable with the nnUNet segmentation, as indicated by the trigeminal nerve highlighted by the yellow arrow in Figure 8. In Figure 9, the segmentation boundaries were compared between manual segmentation and the four deep learning methods, revealing that deep learning boundaries were often smoother than manual segmentation. For each case, the first row represents a two-dimensional slice, the second row represents the three-dimensional rendering of the segmentation volume, and the third row represents the partial three-dimensional rendering.

The average time required for the automatic segmentation with manual modification (performed by radiologists C and D) of 35 test data points was 40.43 ± 7.19 and 35.49 ± 7.28 min, respectively. The average time required for the manual segmentation (performed by radiologists C and D) of 35 test data points was 95.28 ± 7.31 and 95.12 ± 7.33 min, respectively (Table 6). The difference in the average required time between manual segmentation and automatic segmentation with manual modification for both radiologists was statistically significant (*p* < 0.001).

**Table 6:** Comparison of needed time between the manual segmentation and automatic segmentation with manual modification.

Automatic segmentation with manual modification (min)	Manual segmentation (min)	Statistics
35.49 ± 7.28	95.12 ± 7.33	*p* < 0.001
40.43 ± 7.19	95.28 ± 7.31	*p* < 0.001

## 5. Discussion

Trigeminal neuralgia, often referred to as the “king of pain,” poses a significant threat to the physical and mental health of patients (DeSouza et al., 2014). Currently, for trigeminal neuralgia diagnosis, most clinicians primarily focus on the relationship between the nerve and blood vessels in the cistern segment of the trigeminal nerve (Maarbjerg et al., 2014). Nevertheless, some studies have highlighted that abnormalities in the peripheral branches of the trigeminal nerve can also lead to trigeminal neuralgia (Cassetta et al., 2014). With the advancements in magnetic resonance neuroimaging, it has become possible to comprehensively image the trigeminal nerve (Zhang et al., 2021). However, owing to the complex anatomy of the extracranial segment of the trigeminal nerve, the reconstruction process is time-consuming and prone to human error.

The rise of artificial intelligence has led to the application of various deep learning methods in medical image processing. Since AlexNet’s victory in the ImageNet image classification competition in 2012, convolutional neural networks have gained considerable attention due to their superior feature extraction capabilities. This has resulted in the rapid development of three-dimensional medical image processing based on deep learning (Alzubaidi et al., 2021b). Zeng used a three-dimensional U-net network to segment the trigeminal nerve on a three-dimensional FIESTA sequence and segment blood vessels in MRA. By combining the two segmentation results, the relationship between nerve and blood vessel can be automatically recognized (Zeng et al., 2013). Xia et al. proposed a convolutional neural network (Re-NET) based on a reverse edge attention mechanism to achieve three-dimensional cerebral vascular segmentation and surface reconstruction (Xia et al., 2022). Lin et al. used CS2Net to approximately segment the trigeminal nerve and blood vessel, refining their boundaries with three-dimensional UNet to obtain good segmentation results (Lin et al., 2021). Currently, numerous studies focus on deep learning for the trigeminal cistern segment, but applying them clinically remains challenging due to the limited sample size. Deep learning research on peripheral segment branches primarily focuses on the inferior alveolar nerve. For instance, XI et al. employed U-net to segment the inferior alveolar nerve and the third molar on the dental panoramic X-ray films, enabling preoperative evaluation of molar extraction (Vinayahalingam et al., 2019). Lim et al. used a three-dimensional nnU-Net to automatically segment the inferior alveolar nerve on cone-beam CT, achieving a Dice similarity coefficient of (0.58 ± 0.08), indicating relatively general segmentation results (Lim et al., 2021). By contrast, our study is the only one reporting complete automatic segmentation of all three branches of the trigeminal nerve. Compared with previous studies, our study focuses on the entire trigeminal nerve. The SEVB-Net model used in this study demonstrated excellent segmentation performance.

In our study, we explored four approaches: V-Net combined with the *DoubleLoss* method, SEVB-Net combined with the *DoubleLoss* method, nnUNet combined with the *DoubleLoss* method, and SEVB-Net combined with the *ωDoubleLoss* method. Table 3 summarizes the performance of these four models on the testing set. Compared with V-Net and nnUNet, SEVB-Net, which incorporates the bottleneck structure (He et al., 2016), significantly reduced the model’s parameters, resulting in smaller model size, reduced memory usage, and faster forward time. This makes it more suitable for the deployment of network models in cloud or mobile applications in the future. Our results show that, compared with V-Net, nnUNet and SEVB-Net exhibited improved performance in terms of the Dice similarity coefficient (DSC) and sensitivity. Additionally, there was a trend of significant difference in precision between SEVB-Net combined with the *DoubleLoss* method and nnUNet combined with the *DoubleLoss* method (adjusted value of *p* = 0.0972, *p* < 0.1; *d* = 0.7335, *d* > 0.5). Although SEVB-Net did not perform as well as nnUNet in precision, there were no differences in Dice and sensitivity. After replacing *DoubleLoss* with *ωDoubleLoss*, SEVB-Net also outperformed V-Net in precision. Furthermore, there was no significant difference between nnUNet combined with the *DoubleLoss* method and SEVB-Net combined with the *ωDoubleLoss* method, indicating that the trend of a significant precision gap between SEVB-Net and nnUNet was reduced after using *ωDoubleLoss*. Both nnUNet combined with the *DoubleLoss* method and SEVB-Net combined with the *ωDoubleLoss* method achieved a DSC, sensitivity, and precision above 0.7, indicating that the segmentation volumes were in agreement with expert annotations. These results can be attributed to the improved network structure and changes in the loss function. The addition of the bottleneck structure, which includes an extra shortcut branch compared with traditional convolutional structures, allows for deeper network training and mitigates the problem of deep neural network degradation. Furthermore, the SE-Net structure models the correlation between feature channels and strengthens important features to improve accuracy (Hu et al., 2020). Finally, nnUNet exhibits unique advantages in precision, as demonstrated by Ding et al. (2023), Isensee et al. (2021), indicating its potential for complex and fine anatomical structure segmentation. The anatomical complexity of the entire trigeminal nerve poses segmentation challenges. Thicker segments, such as the trigeminal nerve root and ganglion, are easy to segment, whereas the branches of the trigeminal nerve, such as the ophthalmic, maxillary, and mandibular nerves, are prone to under-segmentation due to their thinness. When using the loss function *DoubleLoss*, the network loss is already relatively low when segmenting the thicker trigeminal root, ganglion, and proximal trigeminal branches. The remaining distal trigeminal branches, which are not segmented, have a limited impact on the overall loss, resulting in under-segmentation of the distal trigeminal nerve once the model converges. This limitation can be improved with a larger training dataset, but in cases with limited training data, changing the loss function can improve it to a certain extent. The *ωDoubleLoss* method approximately separates the thicker easily segmented trigeminal nerve root and ganglion from the thinner difficult-to-segment trigeminal nerve branches based on morphological structure. This division results in two regions: the difficult-to-segment region and the easy-to-segment region. By assigning a larger weight to the difficult-to-segment region and a smaller weight to the easy-to-segment region, the loss of the easy-to-segment region is significantly reduced. As the loss in the difficult-to-segment region is relatively high, the model prioritizes optimizing the loss in the difficult-to-segment region.

Owing to the complex anatomy of the whole trigeminal nerve, manual segmentation is a time-consuming process. In this study, both manual segmentation and model-assisted segmentation with manual modification achieved excellent segmentation results but the segmentation process was significantly shorter with the assistance of the deep learning model. Vinayahalingam et al. developed a deep learning method to segment the inferior alveolar nerve and found that model-assisted segmentation with manual modification was 40 s faster than manual segmentation (Vinayahalingam et al., 2019).

In comparison with previous studies, our dataset includes a diverse dataset comprising patients with trigeminal neuralgia and healthy volunteers scanned by four different magnetic resonance scanners with varying field strengths. This diversity in data sources improves the model’s generalization ability. It is well known that learning from a single type of source data can lead to biased outputs tailored to that type of data source. Although the trained model may perform well on that specific data, it may struggle when presented with data from other sources (Hampel et al., 1986). The inclusion of images scanned from multiple devices and the incorporation of data from patients and healthy individuals significantly increases the diversity of input data sources, thereby enhancing generalization ability and model robustness. However, this study also has limitations, primarily stemming from the size of our training dataset, which is relatively small. The minimum dataset size required for effective deep learning varies and is based on multiple factors, including the problem complexity, data diversity, and data quality. In this study, it is recommended that the training sample size should not be less than 100 to achieve desirable segmentation results. According to the power law of deep learning, performance tends to increase with larger datasets (Lei et al., 2019). Data quality and quantity are crucial in deep learning tasks and expanding the training dataset will be essential for further enhancing the model’s performance.

## 6. Conclusion

In conclusion, our results demonstrate that the SEVB-Net model can accurately segment the trigeminal nerve on magnetic resonance three-dimensional volume T2 imaging in patients with trigeminal neuralgia and healthy volunteers. Compared with the basic V-Net model, SEVB-Net enhances segmentation performance, matching nnUNet. It maintains accuracy while offering a more lightweight solution, making it a promising tool for automated trigeminal nerve segmentation, and eliminating manual delineation’s time-consuming obstacle.

## Data availability statement

The raw data supporting the conclusions of this article will be made available by the authors, without undue reservation.

## Ethics statement

The studies involving humans were approved by Medical Ethics Committee of Affiliated Hospital of North Sichuan Medical College (2023ER178-1). The studies were conducted in accordance with the local legislation and institutional requirements. The participants provided their written informed consent to participate in this study.

## Author contributions

CZh: Writing – review & editing, Formal analysis, Funding acquisition, Investigation, Writing – original draft, Methodology, Project administration, Resources. ML: Data curation, Software, Writing – original draft. ZL: Formal analysis, Methodology, Data curation, Writing – original draft. RX: Investigation, Writing – review & editing, Data curation, Methodology. BL: Writing – review & editing. JS: Methodology, Writing – original draft. CZe: Conceptualization, Visualization, Writing – review & editing, Methodology. BS: Investigation, Writing – review & editing. XX: Project administration, Writing – review & editing. HY: Funding acquisition, Project administration, Writing – review & editing.
